# Plasma genistein and risk of prostate cancer in Chinese population

**DOI:** 10.1007/s11255-015-0981-5

**Published:** 2015-05-14

**Authors:** Yishuo Wu, Limin Zhang, Rong Na, Jianfeng Xu, Zuquan Xiong, Ning Zhang, Wanjun Dai, Haowen Jiang, Qiang Ding

**Affiliations:** Department of Urology, Huashan Hospital, Fudan University, 12 Mid-Wulumuqi Road, Shanghai, China; Urology Research Center, Fudan University, Shanghai, China; Shanghai Medical College, Fudan University, Shanghai, China

**Keywords:** Genistein, Plasma, Prostate, Cancer, China

## Abstract

**Objectives:**

Genistein is one of the main soy isoflavones in our daily diet. There were studies proving that high-dietary intake of genistein may relate to the low morbidity and mortality of prostate cancer (PCa) in the Asian population. Since there were few studies of plasma genistein level in the Chinese population, we performed this study to preliminarily evaluate the associations among plasma genistein, epidemiologic factors and PCa in a Chinese population.

**Methods:**

Between 2012 and 2013, 100 men over the age of 40 underwent prostate biopsy for PCa at Huashan Hospital, Shanghai, China. Clinical information, epidemiologic information and blood samples were collected prior to biopsy for each patient. All patients underwent 10-core ultrasound-guided transperineal prostate biopsy, and the pathology results were collected after biopsy. We measured the plasma genistein concentration of the blood samples and analyzed the results along with the clinical and epidemiologic information.

**Results:**

Among the 100 patients, 46 (46.0 %) were diagnosed with PCa. The median plasma genistein concentration of non-PCa patients (728.6 ng/ml) was significantly higher than that of PCa patients (513.0 ng/ml) (*P* < 0.05). In the univariate analysis, we found that age and smoking history were related to PCa (*P* < 0.05). In the multivariate analysis, we found that age, smoking history and plasma genistein were related to PCa (*P* < 0.05). The age-adjusted odds ratio of PCa risk comparing plasma genistein level above median to that below median was 0.31 (95 % CI 0.13–0.71).

**Conclusion:**

Our study suggested that high concentration of plasma genistein level may contribute to the low incidence of prostate cancer in Chinese population.

## Introduction

Prostate cancer (PCa) is the second most common cancer and one of the leading causes of death in men population worldwide [1]. However, the PCa incidence and morbility rate in Chinese population are relatively low compared with that in Western countries. For example, the estimated age-standardized incidence rate (ASIR) of PCa is 145.1 per 100,000 in the USA in 2010, while the ASIR is 12.96 per 100,000 in 2008 in China (unpublished data) [2, 3]. It is suggested that the lower incidence and morbility rate of PCa in Chinese population might be attributable to genetic factors, dietary and environment differences. Considering the possible reasons which caused the differences of incidence and morbility rate of PCa in Chinese and Western populations, preventive measures of PCa had been brought to the attention. The daily diet of Asian population including Chinese contains more soy products, and previous studies had shown that increased soy consumption was associated with a reduced risk of PCa [4–7]. Thus, it made soy food as one of the promising natural chemoprevention against PCa.

Genistein, 4,5,7-trihydroxyisoflavone, is a major soy isoflavone in soy products which are consumed in daily diet. An intake of high-soy products leads to increased blood concentration of genistein, and a number of investigations which included in vitro, in vivo and epidemiological studies on isoflavones including genistein indicated its protective effect against PCa. Asian population who commonly consumes soy products has much higher blood concentrations of genistein than Western population who subsists on a traditional red-meat-based diet.

One of the earliest studies was done among 14 healthy male Japanese and 14 healthy male Finnish to compare the total plasma levels of four soy isoflavones. It was determined that the total plasma level of genistein was approximately 50 times higher in male Japanese than in male Finnish. A hypothesis was made that high plasma levels of soy isoflavones in Japanese were related to the low risk of PCa [8]. Later, a series of studies had investigated the plasma level of genistein in different populations. The different plasma levels of genistein were consistently observed in Asian and Western populations. For example, the plasma levels were about 20 nmol/L in English, 34 nmol/L in Scottish and 260 nmol/L in Japanese and Korean [9–11]. A few studies had also assessed the dietary genistein intake and the connection between soy consumption and PCa risk in Chinese population [4, 12]. However, these studies were mainly based on food frequency questionnaires which might have recall bias, especially in elder people in China. Regarding the limited data of plasma genistein level in Chinese population, we performed this study to preliminarily evaluate the associations among plasma genistein level, epidemiologic factors and PCa in a Chinese prostate biopsy population.

## Methods

### Study population and sample collection

A total of 100 men who underwent prostate biopsy from July 2012 to December 2013 at Huashan Hospital, Fudan University, Shanghai, China, were included as the candidates of the current study. As one of the leading tertiary health institutes in China, patients from all over the country come to Huashan Hospital for medical services.

All the candidates underwent 10-core ultrasound-guided transperineal prostate biopsy. The indications for prostate biopsy at our institute were as follows: (1) tPSA > 4.0 ng/ml; (2) tPSA < 4.0 ng/ml, with suspicious fPSA/tPSA < 0.16 or PSA density [PSAD = tPSA/PV, PV (ml) = height (cm) × length (cm) × width (cm) × 0.52] > 0.15; (3) positive findings from digital rectal examination (DRE), with any level of tPSA; (4) positive findings from imaging techniques such as transrectal ultrasound and magnetic resonance imaging (MRI), with any level of tPSA. All specimens were diagnosed by the Pathology Department of Huashan Hospital. Blood samples before breakfast, and clinical and epidemiologic information (such as age, smoking history and family history) were collected prior to biopsy. Written informed consent was obtained from every patient for their participation in the study. This study was approved by the Institutional Review Board of Huashan Hospital, Fudan University, Shanghai, China.

### Plasma total genistein

All blood samples were measured for total concentration of genistein by a single person in the College of Pharmacy, Fudan University. Plasma (0.25 mL) was treated overnight at 37 °C with 0.75 mL 0.2 mol aqueous ammonium acetate buffer (buffer 1) and 0.5 mL enzyme solution freshly made in buffer 1 which contained about 3000 U β-glucuronidase from Helix (product G7017; Sigma-Aldrich, Saint Louis, MO, USA), 115 U sulfatase from Helix pomatia (product S9751; Sigma-Aldrich, Saint Louis, MO, USA),7.5 mg ascorbic acid to remove glucuronidate and sulfate that are conjugated to genistein by metabolism in the liver. Then, 50 nmol of internal standard 4-hydroxybenzophenone was added and total genistein was extracted with 4 mL methyl tert-butyl ether (MTBE). The MTBE layer was transferred to a silylated glass culture tube and concentrated. The residue was ultrasonic dissolved in 0.3 mL methanol (obtained from Alfa Aesar China (Tianjin, China) Co., Ltd. (Beijing, China)) for 10 min and analyzed with HPLC system.

The HPLC instrumentation is the Waters Alliance high-pressure liquid chromatography system (Waters Corporation, Milford, MA, USA) consisting of an Waters 2489 quaternary solvent delivery system, an Waters 2489 membrane degasser, an Waters 2489 auto-injector, an XBridgeTM C_18_ column (150 × 4.6 mm, 3.5 μm), an C_18_ Guard column (12.5 × 2.1 mm, Agilent Technologies) and a step gradient mobile phase, and genistein was detected with an Waters 2489 UV/visible detector set at 260 nm. Mobile phases consisted of solution A (50 mM aqueous ammonium formate, pH 4.00) and solution B (acetonitrile) at a flow rate of 0.8 mL/min. The gradient program was 0 % solution B for 1 min, increased to 40 % solution B in 0.5 min, held constant at 40 % solution B for 9 min, increased to 80 % solution B in 2 min and ramped down to 0 % solution B in 2.5 min. Genistein concentrations of all samples were calculated through internal standard method by using 0.2 μg/mL genistein as internal standard. Assay-to-assay and day-to-day variability was less than 10 %. The ammonium acetate, ascorbic acid, MTBE and acetonitrile mentioned above were obtained from Sinopharm Chemical Reagent Co., Ltd, Shanghai, China.

### Statistical analysis

In a univariate analysis, the difference in the mean value of age between two groups was tested using the *t* test, the difference in median value of total plasma genistein between two groups was tested using the median test, and the difference in proportions between two groups was tested using a Chi-square test. A stepwise multivariate logistic regression analysis was used to evaluate the independent risks of all clinical and epidemiological variables. A boxplot was used to test the difference in median value of total plasma genistein in a subgroup analysis. A two-sided testing with *P* value of 0.05 was used in the current study. Statistical analyses were performed using SPSS 19.0 (Statistical Product and Service Solutions, IBM Corporation, Armonk, NY, USA).

## Results

The demographic characteristics of the study population are shown in Table 1. Among the 100 patients, 46 (46.0 %) were diagnosed with PCa. The mean age of the whole study population is 70.1 years. The positive rate of family history of cancer and smoking history were 49.0 and 36.0 %. The median total genistein at the time of biopsy were 640.2 nmol/L. The mean age and positive rate of smoking history were statistically higher in men diagnosed with PCa compared with the men without PCa (*P* < 0.05), whereas no significant difference in the positive rate of family history of cancer was observed between PCa and non-PCa groups (*P* > 0.05). The median total genistein at the time of biopsy in PCa group was statistically lower than that in non-PCa group (513.0 vs. 728.6 nmol/L) (*P* < 0.05).

**Table 1:** Epidemiological features and plasma genistein concentration of Chinese prostate biopsy population

Variables	Overall (*n* = 100)	PCa (*n* = 46)	Non-PCa (*n* = 54)	*P* value
Age (year)				
Mean (SD)	70.1 (8.9)	72.5 (8.4)	68.0 (8.8)	0.01^a^
Family history of cancer^d^				
Positive *n* (%)	49 (49.0)	25 (54.3)	24 (44.4)	0.323^b^
Smoking history^e^				
Positive *n* (%)	36 (36.0)	22 (47.8)	14 (25.9)	0.023^b^
Total genistein at the time of biopsy (nmol/L)				
Median	640.2	513.0	728.6	0.005^c^
(IQR)	(255.1–1162.8)	(292.3–901.7)	(229.6–1340.8)	

^a^The *P* values were calculated by using *t* test to see whether there is any significant difference between the means of PCa and non-PCa groups

^b^The *P* values were calculated by using Chi-square test to test whether there is any significant difference between the PCa and non-PCa groups

^c^The *P* values were calculated by using median test of independent sample to test whether there is any significant difference between the PCa and non-PCa groups

^d^Any cancer patient in first-degree relatives was defined as ‘positive’

^e^Patients who had smoked more than 10 cigarettes for a minimum of 10 years was defined as ‘positive’

Then, univariate analysis was performed to test the association between PCa and each variable (age, family history of cancer, smoking history, total genistein at the time of biopsy) **(**Table 2**)**. Age and smoking history were found to be associated with PCa (*P* < 0.05), while family history of cancer and total genistein were not correlated with PCa significantly (*P* = 0.324 and *P* = 0.055). According to the result of univariate analysis, three variables (age, smoking history and total genistein) were included in multivariate logistic regression analysis to test the association between PCa and each variable **(**Table 2**)**. Finally, we found that age, smoking history and total genistein were all associated with PCa (*P* < 0.05). The odds ratios for age, smoking history and total genistein were 1.075, 2.578 and 0.999. These results suggested that elder age and smoking history were risk factors for PCa, while high concentration of total plasma genistein level was a protective factor for PCa.

**Table 2:** Univariate and multivariate analysis of the epidemiological variables at the time of biopsy

Variables	Odds ratio (95 % CI)	*P* value
*Univariate analysis*		
Age	1.065 (1.013–1.118)	0.013
Family history of cancer		
Positive versus negative	1.488 (0.675–3.280)	0.324
Smoking history		
Positive versus negative	2.619 (1.131–6.065)	0.025
Total genistein	0.999 (0.999–1.000)	0.055
*Multivariate analysis*		
Age	1.075 (1.020–1.132)	0.007
Smoking history		
Positive versus negative	2.578 (1.053–6.316)	0.038
Total genistein	0.999 (0.998–1.000)	0.034

Age-adjusted OR and 95 % CI of PCa risk factor for plasma genistein by logistic regression were shown in Table 3. The OR was 0.31 for subjects with plasma level above median to those below median (*P* < 0.05).

**Table 3:** Age-adjusted odds ratio and confidence interval of PCa for plasma genistein by logistic regression among all subjects

	PCa	Non-PCa	Odds ratio	95 % CI	*P* value
Genistein (nmol/L)					
<640.2	30	20	1.00		
>640.2	16	34	0.31	0.13, 0.71	0.006

**Fig. 1:**
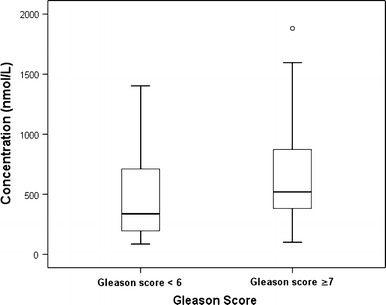
Comparison of the median plasma genistein concentration between PCa patients with Gleason score < 6 and Gleason score ≥ 7 (*P* > 0.05). *PCa* prostate cancer

**Fig. 2:**
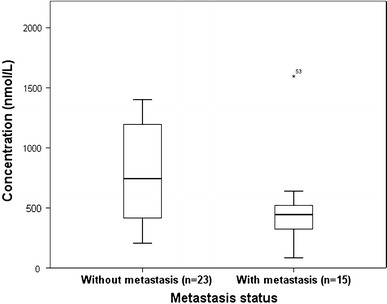
Comparison of the median plasma genistein concentration between PCa patients with metastasis and without metastasis (*P* < 0.05). *PCa* prostate cancer

We also evaluated the relationships between plasma genistein level, PCa patients’ Gleason scores and metastasis status. Of the 46 PCa patients, 45 had their Gleason scores according to their biopsy results. Twelve patients were with Gleason score 6 and 33 with Gleason score ≥ 7. No significant difference was observed in the median total genistein between Gleason score 6 and Gleason score ≥ 7 groups (337.5 vs. 520.0 nmol/L, Fig. 1) (*P* > 0.05). Also, we were able to estimate the metastasis status of 38 PCa patients by their CT scan (or MRI scan) and ECT results within 1 month before or after the biopsy. According to the CT scan (or MRI scan) and ECT results, 15 patients were considered to have metastatic lesions outside prostate, while 23 patients were diagnosed with localized PCa. The median total genistein in metastatic group was statistically lower than that in non-metastatic group (444.8 vs. 744.0 nmol/L, Fig. 2) (*P* < 0.05).

## Discussion

To our knowledge, this study was the first study to examine the association between plasma genistein level, epidemiologic factors and PCa risk in a Chinese population. We firstly described the difference of plasma genistein concentration between PCa and non-PCa subjects. Secondly, we performed univariate and multivariate analysis to test the association between PCa and each variable in the cohort. Thirdly, we performed some subgroup analysis to test whether plasma genistein were associated with tumor malignancy level and metastasis. Overall, a protective effect of plasma genistein against PCa was observed in this study.

Existing studies had already shown that an intake of high-soy products leads to increased blood concentration of genistein [9, 13]. High concentration of genistein was associated with a 70 % reduction in PCa risk in this study. Our results supported previous investigations showing a reduced risk of PCa with high consumption of soy isoflavones in Asian population. A case–control study in China showed a 50 % reduction in PCa risk in the highest soy food and genistein intake tertile [4]. Similar result was also seen in a multiethnic case–control study and a Japanese case–control study [14, 15]. A meta-analysis of the observational studies reported that the combined RR/ORs for soy intake and isoflavone were 0.74 and 0.88. Further separate analysis showed a combined RR/OR of 0.52 from studies with Asian populations and 0.99 from studies with Western populations [16]. However, there were also some studies showing no correlation between plasma isoflavone (including genistein) and PCa [10, 17, 18]. Comparing to the Asian population, the plasma genistein levels reported in the European population (median 34.60 nmol/L for the cases and 33.46 nmol/L for the controls [10], and 25.54 nmol/L for the whole cohort [18]) were much lower. Therefore, one possible reason for the lack of significant correlation observed in the previous European studies is that the relatively low plasma genistein levels were too low for a protective effect to be observed. Besides, the Japanese study considered their inverse results that the higher serum concentrations of isoflavones in the PCa patient group may relate to PCa patients’ increasing consumption of soy food after having been diagnosed with PCa [17]. In our study, we were able to rule out this confounding bias since the patients did not know the biopsy results when collecting the blood samples. As was mentioned in the previous Japanese study, aged people were likely to have higher plasma isoflavone levels which may due to a general change in dietary habits of different generations. Since we chose the prostate biopsy population as our study population which was older overall and had a longtime accumulation of soy food in their diet, it may lead to the result that the plasma genistein levels reported in this study were even higher than that reported in Japanese and Korean population (640 vs. 260 nmol/L). Therefore, it was possible that the plasma genistein levels in this study were high and varied enough to detect the protective effect.

Furthermore, the protective effects of genistein against PCa had already been proved both in vitro and in vivo. The mechanism of genistein against PCa in vivo seemed to involve the following: (1) inhibiting cell invasion by blocking activation of p38 mitogen-activated protein kinase (p38 MAPK) and induction of matrix metalloproteinase type 2 (MMP-2) by transforming growth factor β (TGF-β) [19, 20], (2) inducing the reversion of aggressive endoglin-deficient PCa cells to a low motility phenotype by activating ALK2-Smad1 endoglin-associated signaling [21], (3) inhibiting PCa cell growth by inhibiting Akt kinase and NF-κB signaling [22], (4) regulation of a series of micro-RNAs such as miRNA-151 and miRNA-574-3p [23, 24]. Studies in vitro also showed that: (1) Genistein in diet could reduce the incidence of poorly differentiated adenocarcinoma in transgenic adenocarcinoma of mouse prostate (TRAMP) [25, 26], (2) genistein could inhibit metastasis of human PCa in mice [27], and (3) soy-based dietary may delay PSA progression in PCa patients [28, 29]. Thus, the probable protective effects of genistein in reducing PCa risk and metastatic risk observed in this study were indirectly consistent with these former reported studies.

This study was subjected to several methodological limitations. The one-point blood sampling that we performed in our present study may have bias while representing the subjects’ longtime trend of plasma genistein concentration. However, we had informed all the biopsy patients to maintain their regular diet habits during the period (about 2 week–1 month) before their biopsy in case the sudden changes of diet affected the plasma genistein concentration. Thus, the genistein concentration we obtained in this study could represent the subject’s regular genistein concentration to a certain extent, especially at the time when the subject was diagnosed with PCa or not. Plus, we have included known risk factors in the multivariate regression analysis to minimize the effects of potential confounders. Another limitation is that the sample size in this study was relatively small that we were not able to perform more detailed subgroup analyses by dividing subjects according to different levels of genistein concentration.

Yet we were still able to observe some novel results in this preliminary study: (1) Elder male Chinese had much higher plasma genistein concentrations comparing to previous studies in Western populations, even higher than studies in Japanese and Korean. (2) The median plasma concentration of genistein was higher in non-PCa group than in PCa group. (3) Genistein may have the anti-metastasis effect on PCa. But these findings still needed further discussions in longitudinal follow-up studies in larger Chinese populations.

## Conclusion

Our study suggested that high concentration of plasma genistein level may contribute to the low incidence of PCa in Chinese population.
